# Increased functional activity, bottom-up and intrinsic effective connectivity in autism

**DOI:** 10.1016/j.nicl.2022.103293

**Published:** 2022-12-13

**Authors:** R. Randeniya, I. Vilares, J.B. Mattingley, M.I. Garrido

**Affiliations:** a Queensland Brain Institute, The University of Queensland, Australia; b Department of Psychology, University of Minnesota, USA; c School of Psychology, The University of Queensland, Australia; d Melbourne School of Psychological Sciences, University of Melbourne, Australia; e Canadian Institute for Advanced Research (CIFAR), Canada; f Australian Research Council Centre of Excellence for Integrative Brain Function, Australia

**Keywords:** ASD, Autistic traits, Sensory perceptual, Bayesian models, Prior, Likelihood, Uncertainty, Connectivity, fMRI, DCM

## Abstract

Sensory perceptual alterations such as sensory sensitivities in autism have been proposed to be caused by differences in sensory observation (Likelihood) or in forming models of the environment (Prior), which result in an increase in bottom-up information flow relative to top-down control. To investigate this conjecture, we had autistic individuals (AS) and neurotypicals (NT) perform a decision-under-uncertainty paradigm while undergoing functional magnetic resonance imaging (fMRI). There were no group differences in task performance and in Prior and Likelihood representations in brain activity. However, there were significant group differences in overall task activity, with the AS group showing significantly greater activation in the bilateral precuneus, mid-occipital gyrus, cuneus, superior frontal gyrus (SFG) and left putamen relative to the NT group. Further, when pooling the data across both groups, we found that those with higher AQ scores showed greater activity in the left cuneus and precuneus. Effective connectivity analysis using dynamic causal modelling (DCM) revealed that group differences in BOLD signals were underpinned by increased activity within sensory regions and a net increase in bottom-up connectivity from the occipital region to the precuneus and the left SFG. These findings support the hypothesis of increased bottom-up information flow in autism during sensory learning tasks.

## Highlights

• Autism spectrum group shows increased BOLD activity compared to neurotypicals during a sensory learning task .
• Groups show no differences in BOLD activity for Prior and Likelihood conditions in the sensory learning task.
• Effective hyperconnectivity in bottom-up and intrinsic connections underlies increased functional activity during sensory learning in the autism spectrum group.

## 1 Introduction

Sensory perceptual alterations have been shown to affect over 90% of people on the autism spectrum (Leekam et al., 2007). An understanding of the neural mechanisms that give rise to such perceptual alterations is important for improving diagnostic tools. Bayesian perspectives of atypical perception in autism propose that such perceptual alterations may emerge at the sensory level (likelihood) or from forming poor models (priors) of the environment (Brock, 2012, Pellicano and Burr, 2012, Haker et al., 2016). The high, inflexible precision of prediction errors (HIPPEA) model (Van de Cruys et al., 2014), on the other hand, argues that prediction error weighting is less flexibly adjusted in autism, particularly across different contexts. Increased precision in prediction errors can result in minor deviations from predictions being over-weighted. Van de Cruys (2013) suggest that autism is characterized by an increase in precision in prior variance (i.e., very strong priors) in certain contexts, which are caused by an increase in the precision of prediction errors. Hierarchical Bayesian perspectives, such as the predictive coding view of autism (Teufel et al., 2013, Van Boxtel and Lu, 2013, Lawson et al., 2014, Van de Cruys et al., 2014), further posit that sensory perceptual disruptions such as hypersensitivities may be driven by an increase in bottom-up relative to top-down control in the brain (Van de Cruys et al., 2014, Van de Cruys et al., 2017). When considering the underlying neural processes that give rise to disruptions in sensory learning, such hierarchical models of the brain can provide critical insights (Friston, 2008). Predictive coding, in particular, is a biologically plausible perspective for investigating learning of priors given new sensory evidence (Friston and Kiebel, 2009), and has utility in understanding directed brain networks in autism. In this study, we aimed to understand whether there is an increase in bottom-up information flow in autistic individuals relative to neurotypicals using a Bayesian sensory learning task where we orthogonally manipulated Prior and Likelihood uncertainty.

Increased blood oxygenation level dependent (BOLD) activation in the visual cortex in autism has been reported using a number of sensory perceptual tasks that do not require learning patterns, including the embedded figures test (Lee et al., 2007, Manjaly et al., 2007) and motion perception tasks (Robertson et al., 2014). Such increased visual cortical activation is interpreted as being consistent with increased bottom-up visual processing (Lawson et al., 2014), but it has been unclear if this increase in visual cortical activation is a result of reduced top-down connectivity or only increased bottom-up connectivity. A quantitative *meta*-analysis of functional magnetic resonance imaging (fMRI) of a wide range of basic perceptual tasks in autism (e.g., visual search, visual discrimination) found increased activity in temporal, occipital and parietal regions but decreased activity in the frontal cortex (Samson et al., 2012). More complex tasks that require learning patterns, where engagement of frontal regions is expected, have also shown increased activation in sensory cortices but decreased activation in frontal regions (Clery et al., 2013). Utzerath et al. (2018) used a visual learning task with inanimate (e.g. bike) and animate (e.g. lion) stimuli that would either repeat in pairs or would have an unexpected and less frequent (surprising) stimulus that violated the repetition pattern. They found that adolescents on the autism spectrum showed a typical repetition suppression response, but also differences relative to neurotypicals in fMRI activity in visual area V1 to surprising stimuli (i.e., to stimuli that were less frequent and violated a pattern). Further, a study using a visual change detection paradigm found increased activation in bilateral occipital areas but decreased activation in superior frontal and mid frontal regions in individuals diagnosed with AS relative to NT controls (Clery et al., 2013). Moreover, using a visuomotor task, Villalobos et al. (2005) showed reduced functional connectivity between V1 and inferior frontal cortex in AS individuals relative to controls. While current findings are largely suggestive of increased bottom-up information flow during sensory perceptual tasks, most did not employ tasks designed to explicitly test this, nor did they employ directed connectivity methodology needed to disentangle bottom-up and top-down effects. Indeed, Hadjikhani et al. (2004) suggest that visual cortex organization in adults with AS is typical and differences in visual processing may arise from disruptions in top-down processes instead, which can be evidenced using directed connectivity analysis such as dynamic causal modelling.

In neurotypicals, the orbitofrontal cortex, the amygdala and the putamen have been implicated in forming models of the environment, while the occipital regions have been associated with uncertainty in visual information (Vilares et al., 2012). However, these regions have not been investigated in autism in the context of prior and likelihood uncertainty. In this study, we used a decision-making paradigm (Vilares et al., 2012) in which participants have to decide where a coin fell into a pond. The task manipulates uncertainty in both prior and likelihood, thus enabling the investigation of the Bayesian hypotheses of sensory perceptual disruptions and the underpinning neural representations in autism. This paradigm combined with an effective connectivity approach (i.e., Dynamic Causal Modelling - DCM) also allows us to investigate top-down and bottom-up connectivity that represent priors and likelihoods. Connectivity studies in AS have largely used structural or resting state fMRI techniques, and have reported both under-connectivity and over-connectivity (See Hull et al. (2017) for a review). Thus, it is still unclear which directed brain pathways regulate prior and new information in sensory learning and decision-making in autism spectrum disorder. The overarching aim of the current study was to characterise the brain regions and pathways utilized during sensory learning in autistic individuals, which may provide insights into the hierarchical nature of sensory learning in autism.

## 2 Methods

### 2.1 Recruitment

We recruited 47 neurotypical (NT) adults and 27 adults that reported receiving a diagnosis of an autism spectrum disorder from a clinician. Participants were recruited via autism support groups - Asperger’s Services Queensland, Autism Queensland and Mind and Hearts as well as The University of Queensland (UQ) online recruitment system, UQ newsletter, and online advertisements. Autism spectrum diagnosis was confirmed (for 21 of the 27 participants) by a clinical psychologist using the Autism Diagnostic Observation Schedule for Adults (Gotham et al., 2006, Hus and Lord, 2014). For all group analysis, the autism spectrum (AS) group consisted of 21 autism spectrum confirmed participants and the neuro-typical (NT) group consisted of 21 age- and gender-matched participants. The autistic and sensory sensitivity trait analysis consisted of 74 participants (i.e., 21 AS, 6 participants with an ADOS score <=3 and 47 neurotypical adults). This study was approved by the Human Research Ethics Committee of The University of Queensland (Approval No.: 2019000119).

Participants with a self-reported diagnosis of an autism spectrum disorder undertook an ADOS interview with a trained clinical psychologist. All other stages of the study were the same for NT and AS groups. All participants completed self-report questionnaires and the decision-making task (coin-catching task) while in the MRI scanner.

### 2.2 Questionnaires

Self-report questionnaires such as the Autism Quotient (AQ) questionnaire (Baron-Cohen et al., 2001) and Sensory Perception Quotient (SPQ) Questionnaire (Tavassoli et al., 2014) were used to measure autistic traits and sensory sensitivities, respectively. Participants also completed the Beck Anxiety Inventory (Beck et al., 1988) and Beck Depression Inventory (Beck et al., 1961). Depression and anxiety were measured as they are often comorbidities with autism.

### 2.3 Behavioural task

**Fig. 1:**
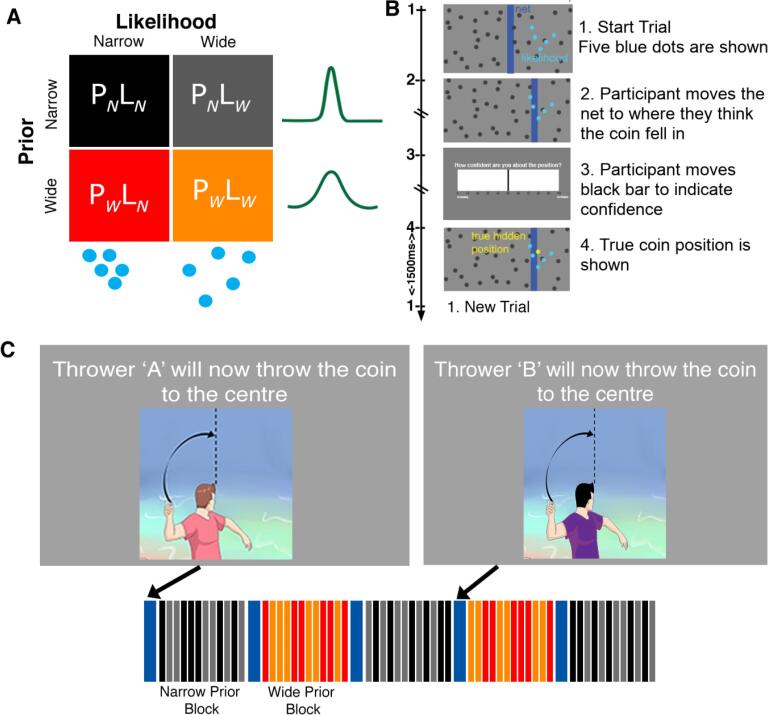
**Coin Task Set up.** A) Task design: the four conditions of the task - with two types of prior (P_N_ = narrow prior; P_W_ = wide prior) and two types of likelihood (L_N_ = narrow likelihood; L_W_ = wide likelihood) uncertainty. B) The time course of a single trial: participants were asked to estimate the position where a coin fell given where the 5 splashes appeared. C) The trials were organized into short blocks with participants being told at the beginning of each block which thrower (“A” or “B”) would be throwing the coin. Four types of trials are shown - Narrow Prior - Narrow Likelihood (P_N_L_N_, black); Narrow Prior - Wide Likelihood (P_N_L_W_, grey); Wide Prior - Narrow Likelihood (P_W_L_N_, red); and Wide Prior - Wide Likelihood (P_W_L_W_, orange). Image reproduced with permission from Randeniya et al. (2021). (For interpretation of the references to colour in this figure legend, the reader is referred to the web version of this article.)

Participants undertook a modified version of the visual decision-making task developed by Vilares et al. (2012), while brain activity was measured in a 3T magnetic resonance imaging (MRI) scanner. Participants were shown an image of a pond on a screen (Fig. 1) and were told that someone was throwing a coin to the middle of the pond (i.e., the middle of the screen). The participants’ task was to move a ‘net’ (blue bar) to where the coin fell in the pond by using a button box. Participants were also asked to rate how confident they were on each trial on a horizontal scale from 0 (guessing) to 100 (very confident); see Fig. 1B. Participants moved a bar on the screen to rate their confidence using the button box. At the end of each trial, participants were shown the true position of the coin, as a yellow dot, for 1500ms. Participants were told there were two different individuals throwing the coin, and that one thrower was better at aiming to the middle than the other. Participants were told which individual (thrower A or B) was throwing at the start of each block, but they were not told which one was the better thrower. The true *likelihood variance* was manipulated *on each trial* by the spread of five blue dots representing the splashes that the coin made when falling into the pond. These dots were taken from a Gaussian distribution where the mean was the coin location in that trial. The spread of the five dots could be narrow (σ_LN_ = 6%) or wide (σ_LW_ = 15%). The prior distribution, from where the coin location was drawn at each trial, had a fixed mean (the center of the screen). The *prior variance* (hidden to the participants) was manipulated across blocks to be narrow (σ_PN_ = 2.5% of the screen width) or wide (σ_PW_ = 8.5% of the screen width), and dependent on the thrower (A and B). Thus, the experiment conformed to a 2x2 design with Prior (wide and narrow) and Likelihood (wide and narrow), yielding 4 types of trials/conditions: *Narrow Prior - Narrow Likelihood* (P_N_L_N_); *Narrow Prior - Wide Likelihood* (P_N_L_w_); *Wide Prior - Narrow Likelihood* (P_w_L_N_); and *Wide Prior - Wide Likelihood* (P_w_L_w_).

***2-Prior Practice Task:*** Before entering the scanner, participants completed a practice of the Coin Task (Fig. 1) on a computer outside of the scanner. The practice task consisted of 2 blocks (one per thrower, 40 trials each). Each block contained an equal number of wide- and narrow-likelihood trials. The practice was 10–15 min in duration.

***Main 2-Prior Task in Scanner:*** The scanner version of the coin task was organized into 24 blocks (12 per thrower/Prior), divided into 4 runs. Before each block began, participants were instructed (for 5 s) which thrower would be throwing next (A or B). Blocks alternated between thrower A and B (narrow and broad prior) and the sequence was counterbalanced across participants. Each block contained 12 trials with 2 types of likelihood (narrow/wide). In total the task consisted of 288 trials, with 72 trials per condition. The task was self-paced and took between 35 and 50 min to complete.

### 2.4 fMRI data acquisition and processing

Participants completed the Coin Task while undergoing whole-brain imaging with a 3-Tesla Siemens Magnetom Trio Tim scanner. Functional MRI data were acquired using a multi-band scanning sequence (TR = 914 ms, TE = 31 ms; FoV = 192 mm; 1.8 mm isotropic resolution; flip angle = 52°). T1-weighted images were acquired with an MP2RAGE sequence with FoV = 256 mm, 176 slices, 1.0 mm isotropic resolution, TR = 4000 ms, TE = 2.91 ms, TI1 = 700 ms, TI2 = 2220 ms, first flip angle = 6°, second flip angle = 7°, and 5 min acquisition time. Functional images were acquired in 4 runs that lasted a maximum of one hour, with some variation due to response times. The visual stimuli were projected on a screen and viewed by the participant through a mirror attached to the head coil. Participants responded using a button box.

Image pre-processing and statistical analyses were performed using Statistical Parametric Mapping (SPM12) software (https://www.fil.ion.ucl.ac.uk/spm), in MATLAB 2019. T1 images of each participant were co-registered to the mean image of each functional volume. All images were normalized to standard MNI template.

### 2.5 fMRI analysis

A standard event-related fMRI approach was used at the subject-specific level of modelling haemodynamic response (first level). For each participant, the onset of each condition was convolved with a canonical haemodynamic response function and regressed against the fMRI signal. The onset times were placed at the beginning of each trial (i.e., when the five blue dots or “splashes” were shown). The first-level general linear model thus contained: a regressor for each condition (P_N_W_N_, P_N_W_W_, P_W_W_N_, P_W_W_W_) per run, six movement (nuisance) regressors per run and the average BOLD response for each run. Excessive movement was detected as participants who had movement spikes greater than the voxel size (i.e., 1.8 mm^3^; for 6 NT and 5 AS) and movement spikes were included in an additional nuisance regressor in the first level GLM. At the group level we conducted a 2 × 2 × 2 ANOVA, with Group (AS, NT), Prior (narrow/wide), and Likelihood (narrow/wide) as factors. For autism trait analysis we conducted a correlation with all participant’s AQ scores and contrasts: 1) Wide Prior > Narrow Prior, 2) Wide Likelihood > Narrow Likelihood.

We also conducted region of interest analyses, based on *a priori* expected regions of interest arising from Vilares et al. (2012). Thus, we used masks for the putamen, insula and amygdala to identify whether there were any between- or within-group effects of the prior, as per Vilares et al. (2012). Masks were generated using the AAL atlas (Tzourio-Mazoyer et al., 2002) in the WFU_pickatlas toolbox (Maldjian et al., 2003) in MATLAB.

We report clusters that survived p < 0.05 family-wise error (FWE) correction at a cluster defining threshold of FWE corrected p < 0.05 or uncorrected p < 0.001 at the whole brain level, using Gaussian random field theory (Worsley et al., 1996). A cluster-defining threshold of p < 0.001 ensures adequate control of cluster-level FWE rates in SPM (Flandin and Friston, 2019).

### 2.6 Dynamic causal Modelling: Specification and motivation

We used dynamic causal modelling (DCM) to investigate the directed connectivity underlying prior and likelihood uncertainty between the two groups, as well as when participants were grouped using AQ scores. Our aim was to understand if there was an increase in bottom-up connectivity in the AS group relative to the NT group, during sensory learning and decision-making. We implemented the Parametric Empirical Bayes (PEB) framework (Friston et al., 2016, Zeidman et al., 2019b, Zeidman et al., 2019a) for DCM in SPM12.

We defined 3 bilateral regions of interest (See Fig. 3A), which included the SFG, precuneus and mid-occipital gyrus identified from the Main Effect of Group contrast (*p*_FWE_ < 0.05). Thus, each participant’s DCM was specified using 6 nodes: left superior frontal gyrus (lSFG; [−22 6 64]), right superior frontal gyrus (rSFG; [18–2 69]), left precuneus (lPreC; [−16–52 60]), right precuneus (rPreC; [10–62 60]), left mid occipital gyrus (lOcc; [−40–70 6]), and right mid occipital gyrus (rOcc;[36–76 26]). The time series BOLD activity for each participant and each node was extracted from an 8 mm radius sphere using an average effect of task contrast of the first level GLM (with an uncorrected threshold of p < 0.001). For each participant the co-ordinate for time series extraction was allowed to move to the nearest local maxima constrained within an anatomical mask of the node generated using WFU_pickatlas toolbox (See Fig. 4A). It was necessary to include the anatomical mask to ensure that the voxels only within regions of interest were included.

**Fig. 2:**
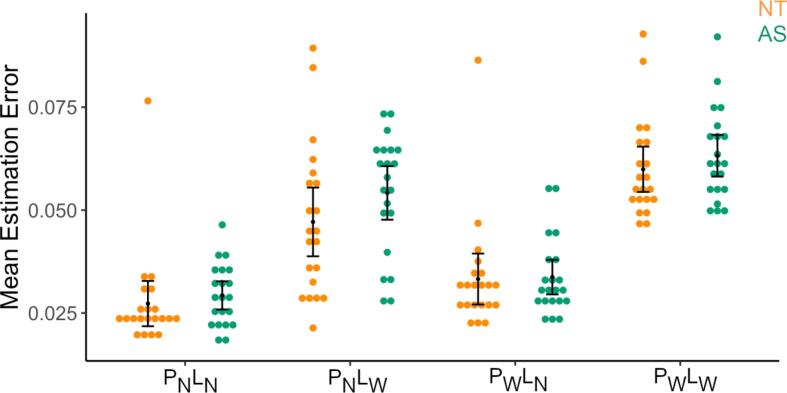
Performance on the coin-toss task, expressed as mean estimation error, was similar for the autistic (AS) and neurotypical (NT) groups.

**Fig. 3:**
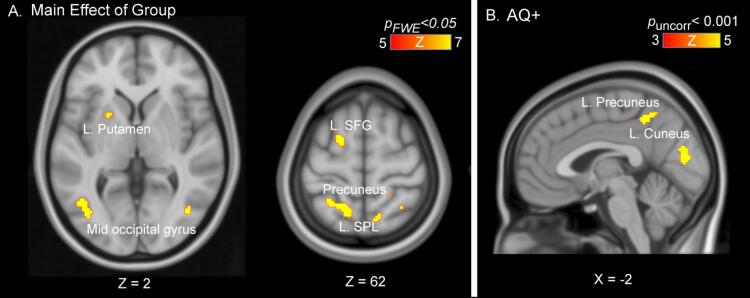
**Group Results.** 2x2x2 ANOVA revealed significant clusters only for A) Main effect of Group (whole brain corrected p_FWE_ < 0.05). B) Activity of effect of likelihood in left precuneus and cuneus were positively correlated with AQ score and effect of wide > narrow likelihood (at whole brain uncorrected p < 0.001, clusters displayed are p < 0.05 cluster-FWE corrected).

**Fig. 4:**
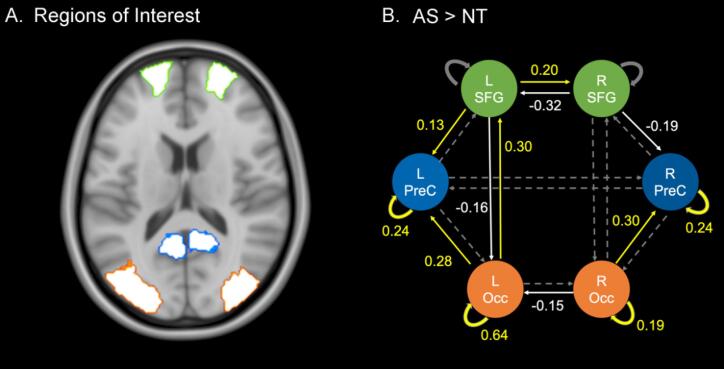
**DCM Results.** A) Regions of interest used to define nodes for DCM models including bilateral precuneus (PreC; blue), superior frontal gyrus (SFG; green) and mid-occipital gyrus (Occ; orange) B) Connection strengths for autism spectrum (AS) group compared with the neurotypical (NT) group. Connections show that the AS group showed increased (yellow) and reduced (white) strength relative to the NT group. Arrows in dashed lines indicate connections that were included in the DCM model but showed no evidence for differences between groups at 95% threshold.

We specified only the A-matrix including all biologically plausible connections (i.e., intrinsic, extrinsic, and lateral connections) in the full model of connectivity based on evidence from published literature, as explained below. A number of studies have found evidence for callosal fibres connecting the bilateral SFG (Briggs et al., 2020), bilateral occipital regions (Dougherty et al., 2005) and bilateral superior parietal lobule including the precuneus (Cavanna and Trimble, 2006). The SFG is connected to the precuneus via the cingulum bundle (Wu et al., 2016, Briggs et al., 2020) and to the superior parietal and occipital regions via the superior frontal occipital fasciculus and inferior frontal occipital fasciculus (Bao et al., 2017).

In order to model the main effect of group we specified only a single regressor, which included the onset of all the trial onsets when specifying a general linear model for each participant’s DCM. Source inputs were specified to the bilateral occipital regions. The full DCM model for each participant was estimated. To understand group connectivity differences in task effects, we used a hierarchical model over parameters implemented by the PEB framework (Friston et al., 2016, Zeidman et al., 2019a). We used PEB as this approach accounts for variability in individual connection strengths and reduces the weight of subjects with noisy data (Zeidman et al., 2019a). We modelled the difference of AS group (1) vs NT group (−1). We report A-matrix connectivity parameters thresholded at a posterior probability of 95 % as in Zeidman et al. (2019a).

## 3 Results

### 3.1 Participants

The final sample had 21 participants in the AS group (Age *M* = 24.38 years, *SD* = 6.37; 12 Females, 8 Males, 1 Intersex) and 21 age-gender matched controls in the NT group (Age *M* = 23.76 years, *SD* = 5.19; 13 Females and 8 Males); see Table 1 for demographic details. Groups showed no differences in their Anxiety [*t* = −0.853, *p* = 0.399] or Depression Scores [*t* = −1.485, *p* = 0.145]. The AS group showed significantly more visual hypersensitivities (i.e., SPQ vision subscale score) compared with the NT group [*t* = 3.270, *p* = 0.002]; see Table 1 for demographic details.

**Table 1:** Demographic Details.

Variable	Neurotypical (NT) Group (*n* = 21)		Autism Spectrum (AS) group (*n* = 21)				Total Sample (*n* = 74) (47 NT + 21 AS + 6 Other)		
	***M***	***SD***	**Range**	***M***	***SD***	**Range**	***M***	***SD***	**Range**
Age (years)	23.76	5.19	18–35	24.38	6.37	18–35	24.38	5.04	18–35
Sex (F/M/Intersex)	13/8/0			12/8/1			39/34/1		
Gender (F/M/Other*)	12/8/1			11/9/1			37/35/2		
Autism Quotient (AQ)	19.10	7.092	4–29	35.43	7.34	21–45	25.47	10.03	4–45
SPQ Total Score	117.10	15.42	90–159	105.62	28.95	58–162	108.24	23.70	50–162
SPQ Vision subscale	29.00	4.01	23–38	23.90	5.91	13–32	25.78	5.67	13–38
Beck Anxiety Score	14.24	12.16	0–55	17.48	12.44	0–44	14.00	11.52	0–55
Beck Depression Score	9.57	7.31	0–26	6.76	1.81	4 – 10	5.84	2.412	2–10
Antidepressant use(Y/N)	0/21			10/11			15/59		
ADHD medication (Y/N)	0/21			6/15			8/66		
ADOS Score	–	–	–	6.76	1.81	4–10	5.85	2.41	2–10

Note: *Other genders include – female to male transgender (1NT & 1 AS).

In the full sample (N = 74; i.e., NT = 47, AS = 21, Other = 6), AQ scores were significantly correlated with the SPQ vision scores [*r* = −0.495, *p* = 7.9 × 10^−5^].

## 4 Behavioural results

### 4.1 No evidence for differences in task performance between groups

A 2 × 2 × 2 analysis of mean estimation error revealed significant main effects of *Prior* [F *=* 33.854*,* η_p_^2^ = 0.458, *p* = 8.506 × 10^−7^] and *Likelihood* [F = 294.217, η_p_^2^ = 0.880, *p* = 4.842 × 10^−20^], with narrower priors and likelihoods being associated with lower estimation errors, but no significant effect of group (*Group* [F = 0.970, η_p_^2^ = 0.024, *p* = 0.330], *Group × Prior* [F = 0.907, η_p_^2^ = 0.022, *p* = 0.347], *Group × Likelihood* [F = 1.796, η_p_^2^ = 0.043, *p* = 0.188]); see Fig. 2. This indicates no difference in task performance between groups and suggests similar task understanding regardless of diagnosis.

Spearman correlation analysis revealed no significant correlation between performance (i.e., mean estimation error) and AQ Scores [*r_s_* = − 0.054, *p* = 0.736, 95 % CI = -0.349 to 0.253] or visual SPQ scores [*r_s_* = 0.004, *p* = 0.981, 95 % CI = -0.318 to 0.337].

We observed a significant Group × Likelihood interaction in confidence reports [F = 10.192, η_p_^2^ = 0.203, *p* = 0.003]). This effect was driven by the NT individuals (M = 6.103), who showed a larger difference in confidence reports for Narrow > Wide likelihood compared with the AS individuals (M = 1.109). There was no significant main effect of *Group* [F = 2.029, η_p_^2^ = 0.048, *p* = 0.162], *Group × Prior* [F = 2.713, η_p_^2^ = 0.064, *p* = 0.107] or *Group × Prior × Likelihood* [F = 0.530, η_p_^2^ = 0.013, *p* = 0.471] interactions.

## 5 fMRI results

### 5.1 Increased BOLD activity in AS group for global task activity

We conducted a 2x2x2 ANOVA with Group (2) × Prior (2) and Likelihood (2) for the fMRI data. There was a main effect of group with significant clusters (p_FWE_ < 0.05, whole brain corrected; Fig. 3A) in the right cuneus [cluster size k_E_ = 287], left mid-occipital gyrus [k_E_ = 203], left precuneus & superior parietal lobule [k_E_ = 209] and left superior frontal gyrus [k_E_ = 80]. Table 2 shows all the significant clusters of activation. The main effect of Prior was not significant, and nor were the Group × Prior interaction, the Group × Likelihood interaction, or the Group × Prior × Likelihood interactions, even at a liberal threshold of p_uncor_ < 0.001.

**Table 2:** Main Effect of Group activation clusters.

**Cluster-level**		**Voxel (Peak-level)**							**Region**
***p*_FWE_**	**k_E_**	***p*_FWE_**	**F**	**Z**	**x(mm)**	**y(mm)**	**z(mm)**	**L/R**	**Region**
3.4 × 10^−12^	287	2.9 × 10^−6^	51.76	6.58	34	−76	8	–	–
		1.2 × 10^−5^	47.76	6.35	28	−82	12	–	–
		2.3 × 10^−5^	45.97	6.24	20	−92	22	R	Superior Occipital gyrus
4.2 × 10^−10^	203	1.2 × 10^−7^	60.47	7.06	−40	−70	6	L	Mid Occipital Gyrus
		8.1 × 10^−5^	42.59	6.02	−46	−64	2		
		0.007	30.93	5.18	−52	−62	−4		
2.9 × 10^−10^	209	1.9 × 10^−6^	52.91	6.65	−16	−52	60	L	Precuneus
		7.1 × 10^−5^	42.97	6.05	−28	−48	58	L	Superior Parietal Lobule
2.3 × 10^−6^	80	0.0004	38.26	5.73	–22	6	64	L	Superior Frontal Gyrus
6.8 × 10^−5^	43	9.5 × 10^−7^	54.93	6.76	–22	−80	26	L	Cuneus
1.3 × 10^−5^	60	2.9 × 10^−5^	45.33	6.20	20	−76	40	R	Superior Occipital Gyrus
2.1 × 10^−5^	55	0.0003	38.90	5.78	10	−62	60	R	Precuneus
2.2 × 10^−4^	32	4.9 × 10^−4^	37.74	5.69	10	−76	50	R	Precuneus
2.2 × 10^−4^	32	0.001	35.77	5.55	–22	8	2	L	Putamen
2.8 × 10^−4^	30	4.7 × 10^−4^	37.87	5.70	34	−48	54	R	Superior Parietal Lobule
3.1 × 10^−4^	29	0.002	34.06	5.43	14	−50	58	R	Precuneus
		0.003	32.72	5.32	20	−44	60	R	Post central gyrus
0.0014	17	0.0002	39.10	5.79	36	−76	26	R	Mid Occipital Gyrus
		0.002	34.64	5.47	−28	14	−2	–	–
0.001	20	1.9 × 10^−5^	48.08	6.37	–22	−90	16	L	Mid Occipital Gyrus
0.002	14	0.002	34.60	5.47	6	−50	26	R	Precuneus
0.003	13	0.0001	41.48	5.95	24	24	30	–	–

We further conducted an analysis adding Anxiety, Depression and Medication-use to the 2x2x2 ANOVA with Group (2) × Prior (2) and Likelihood (2). There was a main effect of group with significant clusters (p_FWE_ < 0.05, whole brain corrected) in the left precuneus and superior parietal lobule, mid-occipital gyrus and left superior frontal gyrus (see Table 3 for details of activation clusters).

**Table 3:** Main Effect of Group Activation Clusters (with Anxiety, Depression and Medication as covariates).

**Cluster-level**		**Voxel (Peak-level)**						**Region**	
***p*_FWE_**	**k_E_**	***p*_FWE_**	**F**	**Z**	**x(mm)**	**y(mm)**	**z(mm)**	**L/R**	**Region**
2.89 × 10^−9^	171	1.80 × 10^−5^	46.86	6.29	−16	−52	60	L	Precuneus
		0.001	35.48	5.53	−28	−54	56	L	Superior Parietal Lobule
3.28 × 10^−5^	50	5.38 × 10^−5^	43.85	6.10	−38	−70	6	L	Mid Occipital Gyrus
4.00 × 10^−5^	48	2.10 × 10^−4^	40.15	5.86	−24	8	4	L	Putamen
6.40 × 10^−4^	23	4.59 × 10^−4^	38.06	5.71	36	−76	10	R	Mid Occipital Gyrus
3.62 × 10^−5^	49	5.05 × 10^−4^	37.80	5.69	12	−62	60	R	Superior Parietal Lobule
7.36 × 10^−5^	42	0.005	31.60	5.23	−24	4	64	L	Superior Frontal Gyrus
		0.011	29.63	5.07	−18	−2	58	–	–

### 5.2 Increased BOLD activity in the cuneus is correlated with autistic traits

When all participants were pooled together, the AQ values were significantly correlated (at whole brain p_uncor_ < 0.001; Fig. 3B) with activity in the left precuneus [r = 0.471, k_E_ = 185, *p*_cluster-FWE_ = 0.002], and the left cuneus [r = 0.462, k_E_ = 166, *p*_cluster-FWE_ = 0.003] for the L_w_ > L_N_ contrast (Fig. 3B). When adjusted for group (i.e., AS or NT as a covariate of no interest), AQ values were correlated only in the left cuneus [r = 0.4214, k_E_ = 159, *p*_cluster-FWE_ = 0.004]. It is important to note than when Anxiety, Depression and Medication were included as covariates to this model, no clusters survived FWE correction. We also conducted a partial correlation analysis with AQ while controlling for SPQ vision. This resulted in no clusters surviving correction.

We found no significant clusters for correlations between AQ or SPQ scores and the P_w_ > P_N_ contrast. SPQ Vision scores showed a correlation with a cluster in the left middle frontal gyrus with L_w_ > L_N_ contrast [r = 0.49 at whole brain p_uncor_ < 0.001, k_E_ = 179, *p*_cluster-FWE_ = 0.002], indicating increased activity in mid-frontal region as hypersensitivities in the sample increased. However, when adjusted for group there were no significant clusters with SPQ vision. Within-group correlations with AQ and SPQ scores did not yield any clusters that survived correction for multiple comparisons.

### 5.3 Within-group validation of Vilares et al. (2012)

Within-group 2x2 analysis of the NT group (N = 47) revealed a significant cluster for the Main Effect of Likelihood driven by Wide Likelihood > Narrow Likelihood (L_w_ > L_N_) in the left mid-occipital gyrus [k_E_ = 177, p_cluster-FWE_ = 0.009, whole-brain p_uncor_ < 0.001]. This replicates findings from Vilares et al. (2012) of likelihood uncertainty represented in occipital regions. However, there were no significant clusters at a similar threshold for the Main Effect of Prior, or for Prior × Likelihood interactions. Nonetheless, a region of interest approach revealed significant clusters at p_uncorr_ < 0.001 for the right putamen [k_E_ = 11, p_cluster-FWE_ = 0.048], right insula [k_E_ = 24, p_cluster-FWE_ = 0.042], and right amygdala [k_E_ = 3, p_cluster-FWE_ = 0.029] for the Narrow > Wide Prior (P_N_ > P_W_) contrast. Within-group analysis of the AS group also did not reveal any significant clusters for the main effect of Prior or Likelihood, either at the whole brain level or with a region of interest approach.

### 5.4 Dynamic causal modelling reveals altered effective connectivity in the AS group

The PEB analysis revealed wide-spread increased effective connectivity in the A-matrix (threshold of 95 %) in the AS group compared with the NT group (Fig. 3B; Table 4). More specifically, we observe increased strength in bottom-up connections from 1) left occipital region to left SFG, 2) left occipital to left precuneus, 3) right occipital to right precuneus as well as in intrinsic connections in the left and right precuneus, and left and right occipital regions. Top-down connections from the left SFG to the left precuneus show an increase in connectivity whereas in 1) left SFG to left occipital and 2) right SFG to right precuneus we observe reduced connectivity strength. We also observe an increase in connectivity from the left SFG to the right SFG and decreased connectivity from 1) the right SFG to the left SFG and 2) right occipital to left occipital regions.

**Table 4:** Effective connectivity strengths in the A-matrix for AS > NT group.

**Connection**	**Connectivity strength at 95 % threshold**
**Top-down connections**	
LSFG → LOcc	(−0.16)
RSFG → RPreC	(−0.19)
LSFG → LPreC	0.13

**Bottom-up connections**	
LOcc → lSFG	0.30
LOcc → LpreC	0.28
ROcc → RPreC	0.30

**Lateral connections**	
LSFG → RSFG	0.20
RsFG → LSfG ROcc → LOcc	(−0.32) (−0.15)

**Intrinsic connections**	
LpreC	0.24
RpreC	0.24
lOcc	0.64
ROcc	0.19

### 5.5 Discussion

We aimed to identify differences in prior and likelihood representations in the brains of autistic and neurotypical adults, and to determine whether such putative differences are also expressed in alterations of directed networks. We did not find group differences in prior and likelihood representations. However, overall group differences for task responses revealed greater activity for the AS group relative to the NT group in the SFG, left putamen, precuneus (+SPL) and occipital regions (mid-occipital and cuneus). The autism trait analysis revealed left lateralized activation in the cuneus as autism scores increased. These findings support previous studies showing increased activity in occipital and parietal regions in autism (Samson et al., 2012, Clery et al., 2013). In contrast to previous studies (Clery et al., 2013, Utzerath et al., 2018), however, we found increased activity in superior frontal gyrus in the autism spectrum group.

Since we only found group differences and no task interaction effects, nor differences in behavioural performance, it is unclear if increased recruitment of the SFG, occipital and putamen regions in the AS group indicate a decrease in efficiency. However, as we observed no group differences in task performance, group differences in recruitment of brain regions and effective connectivity are unlikely to be due to differences in task difficulty between the two groups. The SFG has been implicated in higher order cognition such as working memory as well as in the processing visuo-spatial information (Boisgueheneuc et al., 2006). The precuneus has been implicated in visuo-spatial motor tasks, where memorized patterns and deductive reasoning are necessary (Cavanna and Trimble, 2006). Using resting state fMRI (rs-fMRI) the precuneus (as part of the default mode network) has been shown to have reduced connectivity in autism (Cheng et al., 2015). The putamen has been implicated in learning dependent surprise responses, suggesting a key role in precision setting (Den Ouden et al., 2009). Since we did not observe behavioural differences in overall task performance, and no group differences in prior or likelihood contrasts, we also note that our results may be observing differences in recruitment of the default mode network. It is important to note that we also conducted a validation of the findings of Vilares et al. (2012). Our results replicate their Likelihood findings but are at odds with the direction of uncertainty in the Prior contrast, as we found higher BOLD activity for the Narrow than the Wide contrast in the above-mentioned brain regions. There are several differences between the original paradigm of Vilares et al. (2012) and ours which could have given rise to these differences. Most notably, our task had a higher number of switches between each thrower, which may have increased the difficulty of the learning the task and the prior more specifically. Our sample size was larger than that of Vilares et al., (2012), however, which lends a high degree confidence in our findings.

We also aimed to understand group-differences in effective connectivity that underpin the group differences in BOLD activity for the global task. We modelled only the fixed or endogenous connectivity (A-matrix), which indicate the effectivity connectivity between (and within) regions in the absence of external inputs. Overall, we observe increased effectivity connectivity in the AS group. There is evidence to support (Courchesne and Pierce, 2005, Keown et al., 2013) and refute (Hughes, 2007, Just et al., 2007, Just et al., 2012) that there is widespread hyperconnectivity in the autistic brain. Our findings suggest a more nuanced argument with both hyper- and hypo-connectivity in different pathways. Specifically, we observe *hyperconnectivity* in the AS group (compared to NT) for intrinsic connections in bilateral precuneus and mid-occipital gyrus as well in bottom-up connectivity arising from sensory regions, and from the right SFG to the right Precuneus. The latter is in keeping with a resting-state effective connectivity study that demonstrated increased effective connectivity between prefrontal regions and precuneus (Rolls et al., 2020). Additionally, we found *hypoconnectivity* in top-down connections arising from the left SFG projecting to left occipital node, and from the right SFG to the right Precuneus (note the left to right asymmetry of hypo- and hyperconnectivity across this connection). It is important to note that we have only modelled endogenous effective connectivity, and this does not afford insights into connectivity differences arising from prior or likelihood interactions. Further, increased within-region (intrinsic) connectivity indicates an increase in ‘self-inhibition’. Intrinsic connections modulate excitatory and inhibitory gain between regions, and an increase in these within-region connectivity strengths indicates a reduction in sensitivity to inputs from extrinsic connections.

These findings suggests that the networks in our autistic group are wired for increased bottom-up information flow which is supportive of predictive coding theories of perceptual alterations and sensory overload in autism (Friston et al., 2013, Van Boxtel and Lu, 2013, Van de Cruys et al., 2014).

Our study has several limitations in drawing global inferences relevant to autism. In order to optimize the BOLD signal, we designed a version of the original Vilares et al. (2012) task which had shorter blocks which alternated rapidly. This paradigm change might have made the task relatively difficult for both groups and may thus have resulted in reduced sensitivity to detect differences in prior and likelihood representations between the groups. A further important consideration is the difference in drug use between our two groups. Linke et al. (2017) found that, compared with typically developed children, autistic children and adolescents on psychotropic medication showed underconnectivity between the cerebellum and basal ganglia, but cortico-cortical overconnectivity. In our study the autistic group reported use of anti-anxiety, antidepressant and ADHD medications, the neurotypical group did not report use of any psychoactive drugs. This may be a contributing factor to both improving task performance in some AS participants, as well for the differences in BOLD activity between groups. Studies with a larger sample would be needed to understand the relative contributions of psychotropic medication use in sensory perceptual tasks in autism. Additionally, a significant limitation in interpreting BOLD signal findings from this study is that of possible differences in neurovascular coupling between autistic and neurotypicals. As Reynell and Harris (2013) highlight, neurovascular coupling or oxygen use changes must be experimentally ruled out before BOLD differences can be used as evidence for task-related differences between control and autistic groups and suggest the use of a combination of EEG and fMRI with the same task to rule out such effects. A next step to confirm neuroimaging and DCM findings would be to undertake a control task with simultaneous EEG and fMRI, and to convolve the HRF with EEG signals taking into account neurovascular coupling as in Watanabe et al. (2019).

In conclusion, our results indicate that autistic individuals (relative to matched neurotypicals) have increased recruitment of brain regions during sensory learning and perceptual decision making, but there were no significant differences between the two groups in prior and likelihood representations. Our findings also demonstrate endogenous effective connectivity that may support greater bottom-up information flow in autism. Critically, we show greater neural sensitivity to sensory inputs from both early visual areas and external visual inputs. While providing supportive evidence for previously proposed theories of increased bottom-up information flow in autism, our findings suggest both hyper- and hypoconnectivity alterations in the autistic brain and demonstrate the complexity of the neural mechanisms that underpin autistic sensory learning.

## CRediT authorship contribution statement

**R. Randeniya:** Conceptualization, Data curation, Formal analysis, Visualization, Writing – original draft. **I. Vilares:** Conceptualization, Writing – review & editing. **J.B. Mattingley:** Supervision, Conceptualization, Writing – review & editing. **M.I. Garrido:** Supervision, Conceptualization, Funding acquisition, Writing – review & editing.

## Declaration of Competing Interest

The authors declare that they have no known competing financial interests or personal relationships that could have appeared to influence the work reported in this paper.

## Data Availability

The data has been made available on UQeSpace as Sensory learning in autism: fMRI and DCM Dataset https://doi.org/10.48610/43aebb5.
